# A dual fluorescence reporter system for high throughput screening of effectors of *Kiss1* gene expression

**DOI:** 10.1002/2211-5463.12476

**Published:** 2018-07-03

**Authors:** Xiaoning Li, Sijia Wang, Yanhua Lu, Huanhuan Yin, Junhua Xiao, Kai Li, Lei Ma, Yuxun Zhou

**Affiliations:** ^1^ College of Chemistry, Chemical Engineering & Biotechnology Donghua University Shanghai China; ^2^ State Key Laboratory of Bioreactor Engineering East China University of Science and Technology Shanghai China; ^3^ School of Pharmacy East China University of Science & Technology Shanghai China

**Keywords:** dual fluorescence reporter system, expression effector screening, flow cytometry, high throughput, *Kiss1* gene, kisspeptin

## Abstract

Kisspeptin is a multifunctional peptide encoded by the *Kiss1* gene that plays critical roles in mammalian puberty onset modulation and fertility maintenance in the hypothalamus. Understanding how *Kiss1* expression is regulated is essential for elucidating the molecular mechanisms responsible for these reproductive events. In this study, we constructed an *in vitro* dual fluorescence reporter system to facilitate high throughput screening of effectors influencing the expression of *Kiss1*. In GT1‐7 cells, an enhanced GFP gene was placed under the control of the *Kiss1* gene regulatory elements and translated together with this gene. A tdTomato gene cassette was simultaneously introduced into the same cell for normalization of the fluorescence signal. After treatment with different effectors, the cells were analyzed by flow cytometry. We first tested the efficacy of the system using canonical regulators and then carried out high throughput functional screening to identify chemical compounds that can regulate *Kiss1* gene expression. Of 22 tested compounds from natural sources, 13 significantly affected *Kiss1* expression. Verification by western blot and quantitative reverse transcription PCR (qRT‐PCR) assays and structural analysis identified two chalcone compounds as possible regulators of *Kiss1* gene expression. This system may be suitable for gene functional analysis, drug screening and pharmaceutical studies.

**Abbreviations**
3′‐UTR: 3′‐untranslated region
ARC: arcuate nucleus
AVPV: anteroventral periventricular nucleus
DFRS: dual florescence reporter system
eGFP: enhanced GFP
GnRH: gonadotropin‐releasing hormone
GPR54: G‐protein‐coupled receptor 54
Kp: kisspeptin
mTOR: mechanistic target of rapamycin
qRT‐PCR: quantitative reverse transcription PCR

Kisspeptin (Kp), the product of the *Kiss1* gene, is a multifunctional peptide that plays critical roles in the hypothalamus in modulating the onset of mammalian puberty and maintenance of fertility 1, as well as in some other biological processes outside the brain 2. Kp was first reported for its function in metastasis suppression in malignant melanoma cells in 1996 3, and afterwards, a growing number of studies demonstrated that the level of the *Kiss1* expression was highly relevant to tumorigenesis. In 2001, the hypothalamic Kp was discovered to be a high‐affinity ligand for G‐protein‐coupled receptor 54 (GPR54) by several groups 4, 5, 6. Loss of function of GPR54 was reported as a cause of isolated hypogonadotropic hypogonadism 7, 8, and since then, a large amount of evidence has supported that Kp–GPR54 is the essential gatekeeper of gonadotropin‐releasing hormone (GnRH) neurons, which have a central role in puberty onset and reproduction 9, 10, 11, 12. Kiss1 neurons are involved in a variety of signals that regulate puberty onset, including energy homeostasis, metabolic cues and circadian rhythms, via Kp binding to GPR54 on GnRH neurons 13, 14, 15, 16.

The *Kiss1* gene encodes a 145 amino acid protein that, in rodents, is processed to produce several shorter biologically active C‐terminal amidated products that belong to the Kp family, including Kp52, Kp14, Kp13 and Kp10 (numbers indicating amino acid length) 4. Understanding how the expression of the *Kiss1* gene is modulated in the hypothalamus is pivotal to figure out the molecular mechanism underlying puberty onset regulation. Estradiol (E_2_) is one of the major regulators of the *Kiss1* gene. In GT1‐7 cells, E_2_ treatment increases the expression level of *Kiss1*, while in female mouse hypothalamus, the effect of E_2_ on *Kiss1* expression varies with the anatomy of the Kiss1 neurons 17. The expression of *Kiss1* is inhibited by E_2_ in the arcuate nucleus (ARC), but is stimulated in the anteroventral periventricular nucleus (AVPV) 18. The opposite effect of E_2_ could result from different modes of intracellular E_2_ signaling in the respective regions, with ‘classical’ E_2_ signaling in AVPV Kiss1 neurons and ‘non‐classical’ in ARC Kiss1 cells 1. Moreover, cells around Kiss1 neurons, for example the starvation‐sensitive Agouti‐related peptide‐expressing neurons that attenuate fertility by way of directly inhibiting Kiss1 neurons, proteins in Kiss1 neurons and epigenetic modifications are thought to participate in the regulation of *Kiss1* expression 16, 17, 19. The metabolic factor leptin and the antibiotic rapamycin were confirmed to regulate the expression of *Kiss1* through the mechanistic target of rapamycin (mTOR) pathway 20, 21, 22. Transcription factors EAP1, YY1 and CYX11 were proved to control the transcription of the *Kiss1* gene directly 23. Besides the transcriptional mechanism underlying puberty onset regulation, many other factors, both epigenetic and environmental, also play important roles in this process, and operate through modulating the expression of the *Kiss1* gene 24.

In this study, we constructed an *in vitro* dual fluorescence reporter system (DFRS) to facilitate the high throughput screening of effectors that influenced *Kiss1* gene expression. In immortalized hypothalamic GT1‐7 cells, an enhanced GFP (eGFP) gene was inserted between the coding sequence and the 3′‐untranslated region (3′‐UTR) of the *Kiss1* gene, which shares the same expression regulatory elements with *Kiss1*, visualizing the expression change of *Kiss1* with the help of a fluorescence detector. Simultaneously, we introduced a tdTomato gene to the *Rosa26* locus in the same cell to have the cell emit red fluorescence, together with green fluorescence. After normalization by the intensity of red fluorescence, the change of green fluorescence can be compared among cells challenged by different effectors, such as chemical compounds, microRNAs (miRNAs), cytokines and protein‐expressing plasmids. With the help of a flow cytometer, we tested the efficacy of the system with E_2_, rapamycin and miRNAs, and then carried out functional screening to identify chemical compounds that could regulate the expression of the *Kiss1* gene. Supported by western blot, quantitative reverse transcription PCR (qRT‐PCR) assay and structural analysis, two chalcone compounds were deemed to be potential regulators of *Kiss1* gene expression.

## Materials and methods

### Vector construction

To generate CRISPR plasmid ‘42230‐Kiss1’ expressing both cas9 endonuclease and the chimeric single guide RNA (sgRNA), a 20 bp protospacer sequence of the *Kiss1* gene was designed according to the website http://crispr.mit.edu/and cloned into the pX330‐U6‐Chimeric_BB‐CBh‐hSpCas9 (Addgene, Cambridge, MA, USA; 42230) plasmid at a *Bbs*I site 24. For generation of a Kiss1‐2A‐eGFP GT1‐7 reporter line, we carried out a selection‐free knockin strategy as previously described 25. The pKiss1‐2A‐eGFP donor plasmid was constructed using the one‐step directed cloning kit (Novoprotein, Shanghai, China) as follows. First, a pair of oligonucleotides (2A‐eGFP‐F and 2A‐eGFP‐R) containing a P2A sequence were annealed, extended by Taq DNA polymerase and cloned into *Age*I‐digested FUGW vector in front of the eGFP gene. Then three DNA fragments, the 594 bp left homology arms of the *Kiss1* gene (primer: F1 and R1, PCR amplified from GT1‐7 genome), the P2A‐eGFP coding sequence (primer: F2 and R2, PCR amplified from FUGW‐2A‐eGFP) and the 900 bp right homology arms of the *Kiss1* gene (primer: F3 and R3, PCR amplified from GT1‐7 genome) were cloned in the expected order into *Eco*RI (New England Biolabs, Ipswich, MA, USA)‐ and *Bam*HI (New England Biolabs)‐digested pUC19 simultaneously according to the manufacturer's instruction. The resulting plasmid, pKiss1‐2A‐eGFP, carried a 2A‐eGFP sequence between the codon of the last amino acid and the stop codon of the *Kiss1* gene.

For generation of the reference red fluorescence signal, Ai9‐tdTomato was modified from Ai9 26 by cutting the stop signal between two flox sequences using Cre recombinase (New England Biolabs).

The DNA sequences for all oligonucleotides used for the generation of the above vectors are listed in Table 1.

**Table 1:** List of all oligonucleotides and primers

Oligo or primer name	Sequence (5′→3′)
42230‐Kiss1‐sgRNA‐F	CACCGCAGGCGGCGCGGGCAGCACG
42230‐Kiss1‐sgRNA‐R	AAACCGTGCTGCCCGCGCCGCCTGC
2A‐eGFP‐F	ATCCCCGGGTACCGGTGCTAGCGCCACTAACTTCTCCCTGTTGAAACAAGCAGGGGATG
2A‐eGFP‐R	TCCTCGCCCTTGCTCACCATTGGCCCGGGATTCTCTTCGACATCCCCTGCTTGTTTCAA
F1	GACGGCCAGTGAATTCCGGTCCCTCCTTTTGCTCTT
R1	CGACTCTAGAGGATCCGCCCCGTGCTGCCCGCGCCG
F2	CGGGCAGCACGGGGCGCTAGCGCCACTAACTTCTC
R2	CGACTCTAGAGGATCCTTACTTGTACAGCTCGTCCA
F3	GAGCTGTACAAGTAAGTGCTGGGCTGCAGGTGGAT
R3	CGACTCTAGAGGATCCTCTGTGTGTTCAAAGCCAGC
Test‐primer‐F1	GCTCAGACTCCAGACACACT
Test‐primer‐R1	GGTGTTCTGCTGGTAGTGGT
Test‐primer‐F2	CACATGAAGCAGCACGACTT
Test‐primer‐R2	ATCTTGGGGTTGGGAATGGT
Kiss1‐2A‐eGFP‐F	CCACCTACAACTGGAACT
Kiss1‐2A‐eGFP‐R	CCGGTGAACAGCTCCTCG
Kiss1‐F	CTTCTCCTCTGTGTCGCCA
Kiss1‐R	TACCGCGATTCCTTTTCCCA
GAPDH‐F	CCCACTCTTCCACCTTCGATG
GAPDH‐R	CCACCACCCTGTTGCTGTA
Kiss1‐WT‐3′UTR‐F	TAGGCGATCGCTCGAGGTGCTGGGCTGCAGGTGGATTGTAGAGGCCAAGGCAGGGAGCT
Kiss1‐WT‐3′UTR‐R	TTGCGGCCAGCGGCCGCGACGGCAGCATTGCTTTTATTGCACAAGTCTAGAAGCTCCCT
Kiss1‐MU‐3′UTR‐F	TAGGCGATCGCTCGAGGTGCTGGGCTGCAGGTGGATTGTAGAGGCCAAGGCAGGGAGCT
Kiss1‐MU‐3′UTR‐R	TTGCGGCCAGCGGCCGCGACGGCAGCATTGCTTTTAGATATCAAGTCTAGAAGCTCCCT

### Cell culture and transfection

GT1‐7 and human embryo kidney (HEK) 293 cells were cultured in high‐glucose Dulbecco's modified Eagle's medium (Key GEN BioTECH, Nangjing, China), containing 10% FBS (Thermo Fisher Scientific, Waltham, MA, USA) and 100 U·mL^−1^ penicillin–100 μg·mL^−1^ streptomycin in a humidified 5% CO_2_ incubator at 37 °C.

Transient transfection of plasmids and miRNA mimics (GenePharma, Shanghai, China) were performed using Lipofectamine™ 2000 reagent (Thermo Fisher Scientific) according to the manufacturer's recommendations. Cells were seeded into 24‐well plates 1 day prior to transfection at a density of 100 000 cells per well. For eGFP targeting, 1 μg of 42230‐Kiss1 and 1 μg of pKiss1‐2A‐eGFP were used. For tdTomato targeting, 1 μg of Ai9‐tdTomato was used. For a luciferases gene reporter assay, 20 pmol of miRNA mimics and 1 μg of wild‐type or mutant psicheck2‐Kiss1‐3′UTR were used.

### Flow cytometry detection

For generation of the Kiss1‐2A‐eGFP GT1‐7 reporter line, cells were transfected with 42230‐Kiss1 and pKiss1‐2A‐eGFP and then incubated for 48 h in a 24 well‐plate. Then they were seeded into a T75 flask. After 15 days, cells were resuspended at a density of 10^7^ cells·mL^−1^, digested by trypsin and sorted with a flow cytometer (MoFlo XDP; Beckman Coulter Life Sciences, Indianapolis, IN, USA). The eGFP positive cells were sorted with the single‐cell module. The collected cells were cultured in 5% CO_2_ at 37 °C for propagation and further detection.

Flow cytometry was also applied in detecting the change of fluorescence intensity quantitatively. After treatment with β‐estradiol (Sigma‐Aldrich, St Louis, MO, USA), rapamycin (Sigma‐Aldrich), miRNAs (Gene Pharma, Shanghai, China) or chemical compounds for 24 h, cells were digested by trypsin and resuspended in 4% paraformaldehyde. After 30 min fixing on ice, 10 000 cells that were similar in morphology from each sample were measured for fluorescence intensity by with a BD FACSCalibur (Becton, Dickinson and Company, Franklin Lake, NJ, USA). The geometric means of fluorescence intensity of eGFP and tdTomato were used for comparison between samples.

### Genotyping for P2A‐eGFP targeting

**Figure 1:**
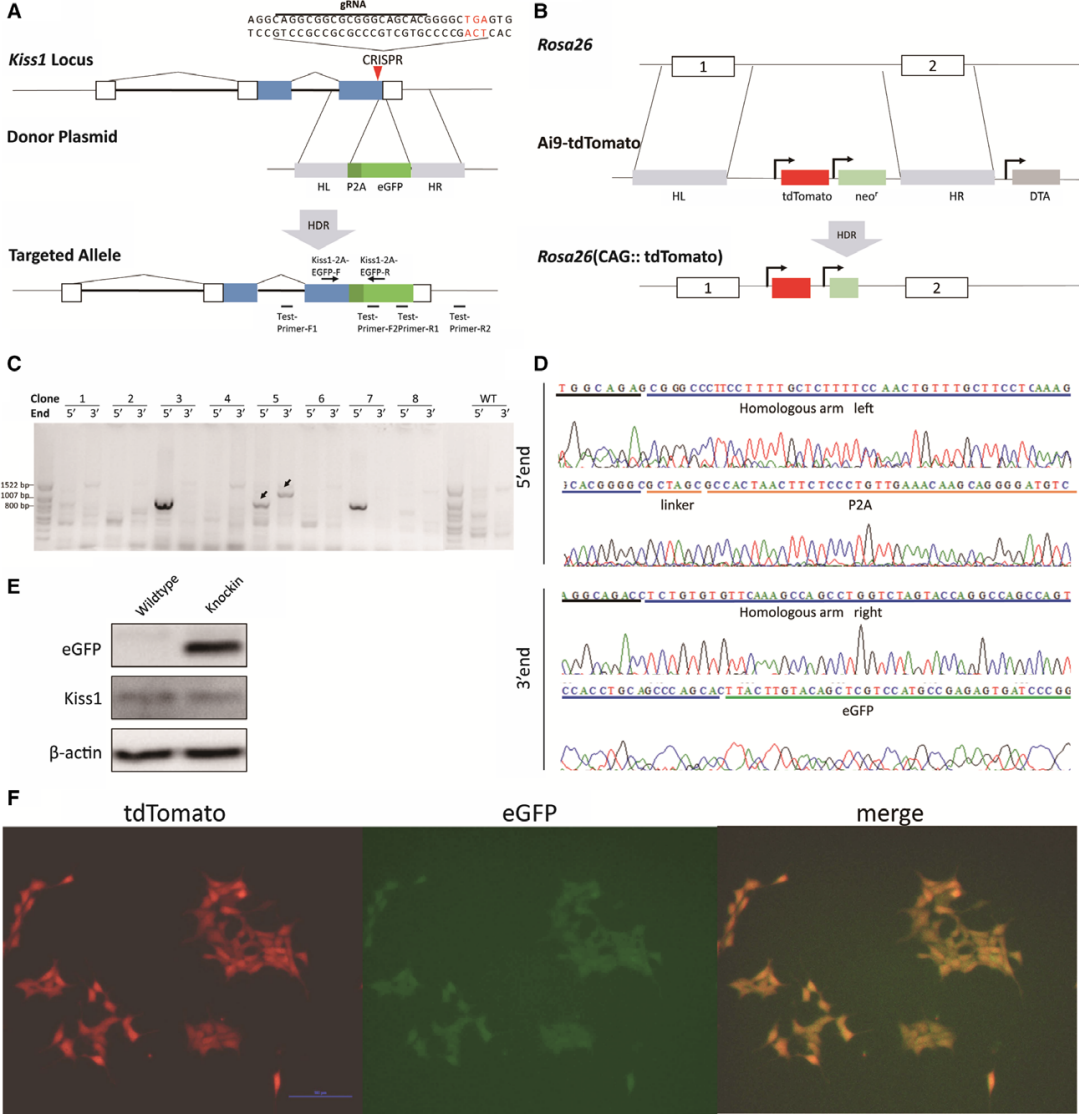
Construction of DFRS for *Kiss1* expression. (A) Schematic representation of the targeting strategy of P2A‐eGFP for the *Kiss1* locus. In the presence of the donor plasmid, homologous directed recombination results in the insertion of P2A‐eGFP into the *Kiss1* gene between the last amino acid codon and the stop codon. The white boxes represent the non‐coding exons of *Kiss1* and the blue represent the coding sequence. The thick lines indicate the intron of *Kiss1*. The PCR primers for detection used for genotyping are indicated with bars (PCR product length: test primer F1+ test primer R1 = 774 bp; test primer F2+ test primer R2 = 1064 bp). The qRT‐PCR primers used for detecting the co‐expression of *Kiss1* and eGFP are indicated with arrows. (B) Schematic representation of the targeting strategy for tdTomato. In the presence of the donor plasmid, homologous directed recombination results in the insertion of tdTomato between exon 1 and exon 2 of *Rosa26*, which are indicated with white boxes. (C) The result of genotyping of P2A‐eGFP targeting by PCR. No. 5 is the correctly targeted clone as both 5′ arm and 3′ arm can be PCR amplified. Arrows point to the PCR products with correct length. (D) Sequencing results of the correctly targeted allele in the Kiss1‐P2A‐eGFP reporter line. (E) Western blot analysis for Kp and eGFP in the Kiss1‐P2A‐eGFP reporter line with correctly targeted allele. (F) Fluorescence microscopy image displaying the co‐expression of eGFP and tdTomato in GT1‐7‐Kiss1‐2A‐eGFP‐Rosa26 (CAG::tdTomato) cell line. Scale bar: 100 μm.

To confirm the homologous recombination events on the *Kiss1* locus in GT1‐7 cells, the targeted regions were PCR amplified, templated by the genomic DNA of eGFP positive clone. The location of primers and the size of their respective products are shown in Fig. 1 and the primer sequences are listed in Table 1. PCR products were separated on 1% agarose gel, and the products with correct length were confirmed by DNA sequencing.

### qRT‐PCR

For gene expression analyses, total RNA from the reporter cell line was extracted using RNAiso Plus (Takara Bio Inc., Kusatsu, Japan) according to the manufacturer's recommendations. Genomic DNA was eliminated with DNase I (Thermo Fisher Scientific), then the cDNA was synthesized with the Thermo Fisher Scientific RevertAid First Strand cDNA Synthesis Kit with oligo‐dT as primers. qRT‐PCR was carried out with an Applied Biosystems 7500 Real‐Time PCR system using SYBR Green SuperReal PreMixPlus (Tiangen, Beijing, China), and the primers for each gene are listed in Table 1.

### Western blot

The cells to be analyzed by western blot were seeded into six‐well plates with 5 × 10^4^ cells per well, cultured until confluence and then treated by different effectors. After removal of the medium, the cells were washed twice with ice‐cold PBS, then lysed using 100 μL RIPA (Beyotime, Shanghai, China). The lysates were centrifuged at 12 000 ***g*** at 4 °C for 10 min. The samples were boiled for 5 min and loaded onto a 4% SDS/PAGE for electrophoresis for 30 min at 80 V and 60 min at 120 V in 12% SDS/PAGE. Then the protein was transferred onto 0.2 μm pore‐size poly(vinylidene difluoride) membranes (Merck Millipore, Billerica, MA, USA) at 280 mA for 90 min. The membranes were blocked at room temperature for 1 h with blocking solution (5% BSA in TBST), and incubated overnight at 4 °C with the appropriate primary antibody diluted in blocking buffer (rabbit polyclonal anti‐eGFP, 1 : 1000, CAB4211, Thermo Fisher Scientific; goat polyclonal anti‐Kiss1, 1 : 500, sc‐18134; Santa Cruz Biotechnology, Dallas, TX, USA; mouse monoclonal anti‐β‐actin, 1 : 1000, sc‐47778; Santa Cruz Biotechnology). The membranes were washed three times with TBST before being exposed to horseradish peroxidase‐conjugated secondary antibody (anti‐rabbit IgG, anti‐goat IgG, anti‐mouse IgG, respectively, 1 : 1000; Beyotime) diluted in blocking buffer for 1 h at room temperature. Immunoreactions were visualized using an ECL detection kit (Thermo Fisher Scientific). Immunoblots were scanned and analyzed using image lab software (Bio‐Rad, Hercules, CA, USA).

### Dual luciferase gene reporter assay

To generate the dual luciferase gene reporter of *Kiss1*, two pairs of oligonucleotides (Kiss1‐WT‐3′UTR‐F and Kiss1‐WT‐3′UTR‐R; Kiss1‐MU‐3′UTR‐F and Kiss1‐MU‐3′UTR‐R; the sequences are listed in Table 1) containing full‐length 77 bp wild‐type or mutant 3′‐UTR sequence of the *Kiss1* gene were annealed, extended by Taq DNA polymerase and cloned into the *Not*I–*Xho*I site of the psiCHECK‐2 vector (Promega, Madison, WI, USA) using the one‐step directed cloning kit. The vector containing the wild‐type 3′‐UTR sequence of the *Kiss1* gene was named Kiss1‐3′UTR‐WT. The vector carrying mutations in the seed region of the miRNA binding site in the 3′‐UTR sequence of the *Kiss1* gene with GUGCAAU replaced by GAUAUCU was named Kiss1‐3′UTR‐MU.

The luciferase assays for miRNAs were performed as previously described 27. Briefly, 5 × 10^4^ HEK 293 cells were plated in 24‐well plates and after incubation for 48 h, transfected with either miRNA mimics or scramble and wild‐type or mutant *Kiss1* psiCHECK‐2 vector (Promega). Forty‐eight hours later, cells were lysed using Reporter Lysis Buffer, and *Renilla* or firefly luciferase expression was analyzed using a Dual Luciferase Reporter Gene Assay Kit (Beyotime) according to the manufacturer's recommendations.

### Chemical compound screening

To screen chemical compounds affecting *Kiss1* expression, the cell line were seeded into 24‐well plates with 2 × 10^4^ cell per well and cultured until confluence; 2.5 μL of 2 mm compounds diluted with DMSO was added into the culture medium at 10 μm final concentration. After incubated for 24 h, the cells were prepared for the flow cytometry assay.

### Statistics

All data analyses of qRT‐PCR and flow cytometry were performed using prism 5 (GraphPad Software, La Jolla, CA, USA). Data were compared using a non‐parametric paired two‐tailed Student's *t* test. Western blot data were analyzed using Image Lab (Bio‐Rad).

## Results

### The construction of a DFRS for *Kiss1* expression

In order to exhibit the expression level of *Kiss1* by a reporter gene synchronously and faithfully, we carried out gene targeting in hypothalamic GT1‐7 cells, inserting a P2A‐eGFP coding sequence in‐frame in the *Kiss1* gene between the last amino acid codon and the stop codon, so as that the eGFP shared the full set of regulatory elements with the *Kiss1* gene. P2A is a self‐splicing spot in the resulting fusion protein 28 (Fig. 1A). We kept eGFP positive clones screened by fluorescence activated cell sorting individually for further culture and detection. We identified one correctly targeted clone by PCR from 21 cultures and confirmed the results by DNA sequencing (Fig. 1C,D). The separated protein products of Kp and eGFP were detected in the targeted cells: GT1‐7‐Kiss1‐P2A‐eGFP (Fig. 1E).

In addition, we introduced a red fluorescent protein, tdTomato, gene as a reference signal into GT1‐7‐Kiss1‐P2A‐eGFP cells by Ai9‐tdTomato transfection to normalize the non‐specific influence on the protein synthesis of the cell (Fig. 1B). After drug selection, tdTomato + G418‐resistant clones, GT1‐7‐Kiss1‐2A‐eGFP‐Rosa26 (CAG::tdTomato), were retained as a dual fluorescence reporter for *Kiss1* expression (Fig. 1F).

### The efficacy of the DFRS of *Kiss1* expression tested by canonical regulators

**Figure 2:**
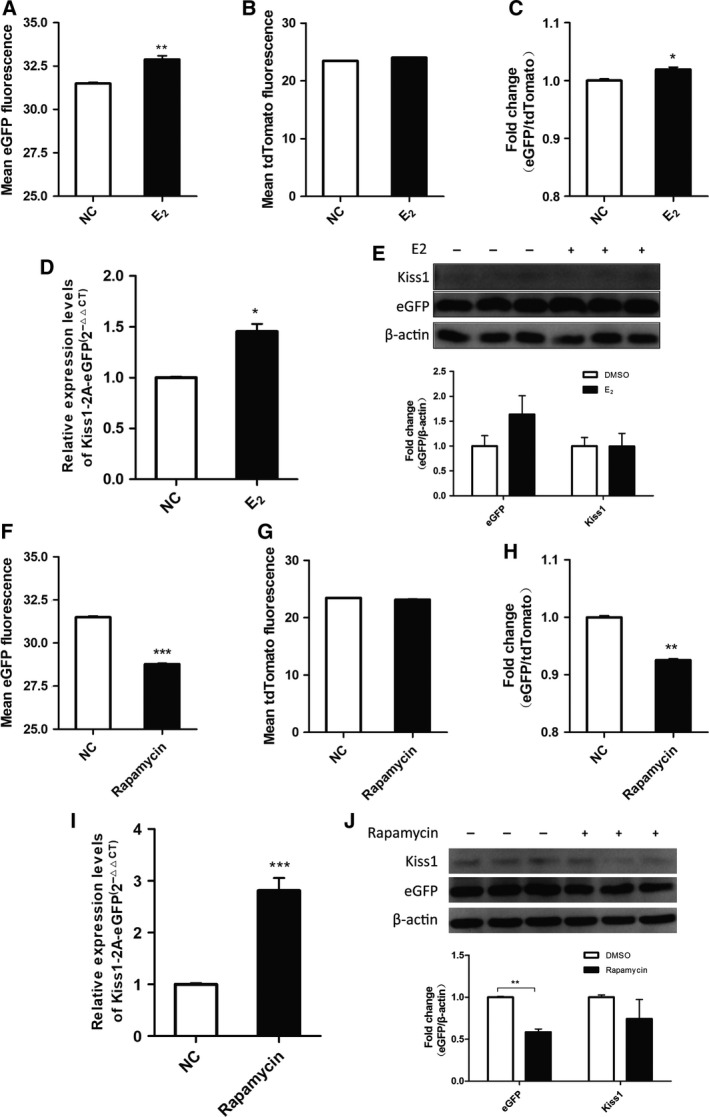
DFRS of *Kiss1* expression responds to *Kiss1* regulators. (A,B) Mean fluorescence intensity of eGFP and tdTomato of the reporter treated with E_2_ (*t* test, E_2_ vs negative control (NC), *P*_e_ _GFP_ = 0.0040, *n* = 3, **P <* *0.01). (C) Fold change of normalized eGFP/tdTomato of the reporter treated with E_2_ (*t* test, E_2_ vs NC,* P* = 0.0352, *n* = 3, *P <* *0.05). (D) Relative mRNA expression level of *Kiss1* and eGFP of the reporter treated with E_2_ (*t* test, E_2_ vs NC,* P* = 0.0114, *n* = 3, *P < 0.05). (E) Relative protein expression level of *Kiss1* and eGFP of the reporter treated with E_2_ (*t* test, E_2_ vs NC,* n* = 3, *P*_e_ _GFP_ = 0.3766, *P*_K__iss1_ = 0.9758). (F,G) Mean fluorescence intensity of eGFP and tdTomato of the reporter treated with rapamycin (*t* test, rapamycin vs NC,* n* = 3, *P*_e_ _GFP_ = 0.0001, ***P < 0.001). (H) Fold change of normalized eGFP/tdTomato of the reporter treated with rapamycin (*t* test, rapamycin vs NC,* n* = 3, *P* = 0.0038, **P < 0.01). (I) Relative mRNA expression level of *Kiss1* and eGFP of the reporter treated with rapamycin (*t* test, rapamycin vs NC,* n* = 3, *P* = 0.0007, ***P < 0.001). (J) Relative protein expression level of *Kiss1* and eGFP of the reporter treated with rapamycin (*t* test, rapamycin vs NC,* P*_e_ _GFP_ = 0.0069, *P*_K__iss1_ = 0.3843, *n* = 3, **P < 0.01). All values shown are means ± SEM.

To evaluate the efficacy of the DFRS for the *Kiss1* gene, we inspected the fluorescence intensity variation quantitatively in cells with E_2_ or rapamycin treatment by flow cytometry, as estrogen was proved to be an activator and rapamycin a repressor of *Kiss1* expression in previous work 17, 21. After incubated with 100 nm E_2_ or 70 nm rapamycin for 24 h, the cells were trypsinized and loaded into the flow cytometer. The intensities of green and red fluorescence of 10^4^ cells for each sample were recorded. The green fluorescence intensity grew stronger with E_2_ treatment, but became weaker with rapamycin treatment (Fig. 2A,F). The red fluorescence intensity showed no significant differences in either experimental sample (Fig. 2B,G). Given that tdTomato was predesigned as an internal control in this system, the value of eGFP/tdTomato was introduced to represent the normalized expression of eGFP. The normalized eGFP/tdTomato value of cells treated by E_2_ and rapamycin also displayed significant differences (Fig. 2C,H). The results of the E_2_ treatment from flow cytometry detection were confirmed by a qRT‐PCR assay (Fig. 2D) and western blot (Fig. 2E). The result of the rapamycin treatment from flow cytometry detection were contrary to that determined by qRT‐PCR (Fig. 2I), but consistent with that determined by western blot (Fig. 2J).

**Figure 3:**
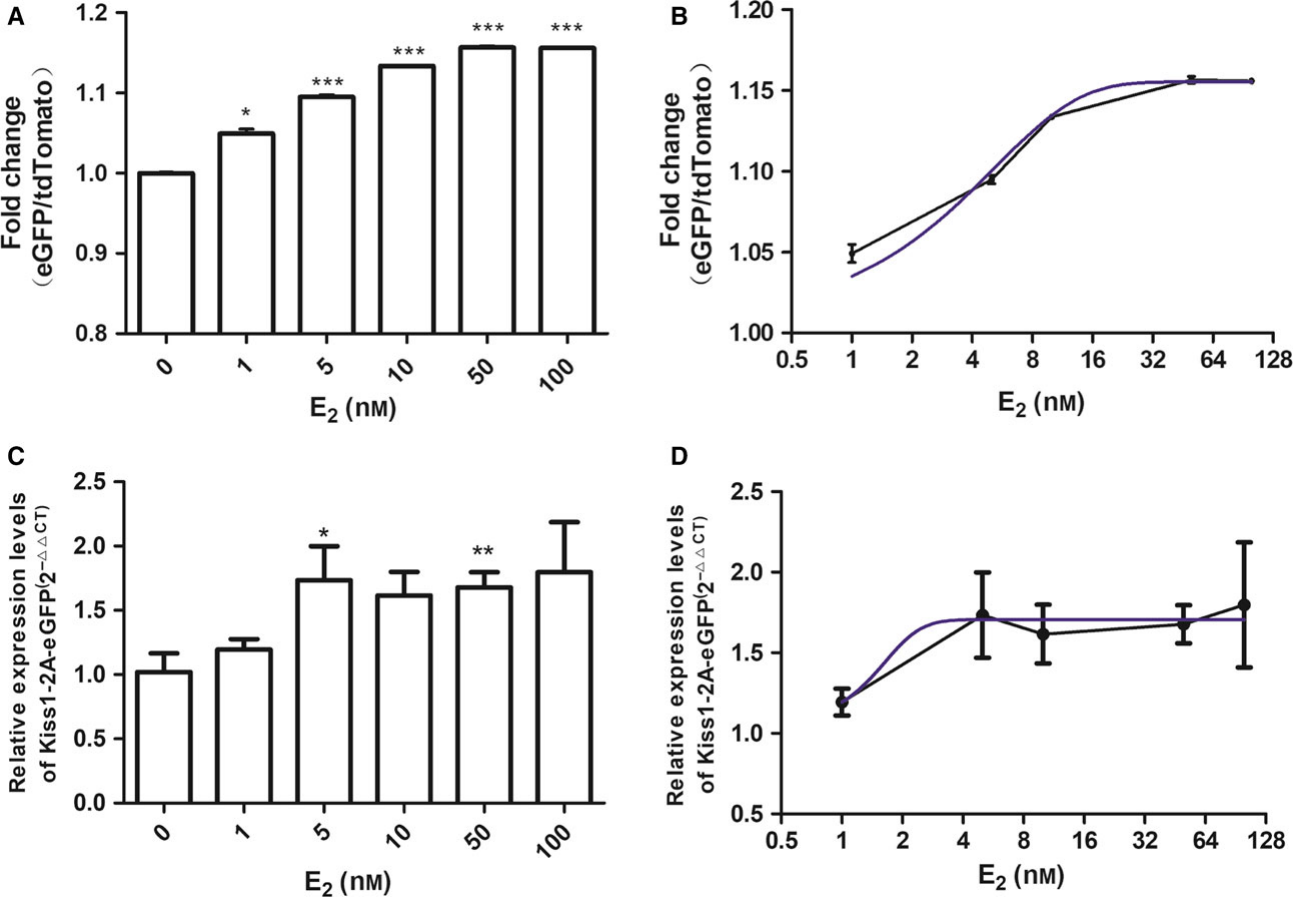
Dose responses of the *Kiss1* reporter treated with E_2_. (A) Dose responses of the *Kiss1* reporter treated with E_2_ detected by flow cytometry (*t* test, every group vs E_2_ = 0 nm,* P*_1_ = 0.0122, *P*_5_ = 0.0005, *P*_10_ < 0.0001, *P*_50_ = 0.0002, *P*_100_ = 0.0001, *n* = 3, *P <* *0.05, ***P <* *0.001). (B) Dose–response curve of the *Kiss1* reporter treated with E_2_ detected by flow cytometry (non‐linear fit, variable slope, *R* ^2^ = 0.9799). (C) Dose responses of the *Kiss1* reporter treated with E_2_ detected by qRT‐PCR (*t* test, every group vs E_2_ = 0 nm,* P*_1_ = 0.2445, *P*_5_ = 0.0448, *P*_10_ = 0.2065, *P*_50 _= 0.008, *P*_100 _= 0.2804, *n* = 3, *P <* *0.05, **P <* *0.01). All values shown are means ± SEM. (D) Dose–response curve of the *Kiss1* reporter treated with E_2_ detected by qRT‐PCR (non‐linear fit, variable slope, *R* ^2^ = 0.4472).

To determine the reliability and sensitivity of the dual fluorescence reporter of *Kiss1* expression, we performed a dose–response test with serial dilutions of E_2_. We investigated the outcome of the treatment by means of qRT‐PCR and flow cytometry. The dose–response effect could be observed with all of the detection methods. Nevertheless, compared with the results of qRT‐PCR, our reporter system showed better dose‐dependent responses by flow cytometry (Fig. 3). The correlation coefficient, *R* ^2^, of normalized eGFP/tdTomato reached 0.9799 (Fig. 3B).

### miRNAs regulating the expression of *Kiss1* gene directly confirmed by the DFRS

**Figure 4:**
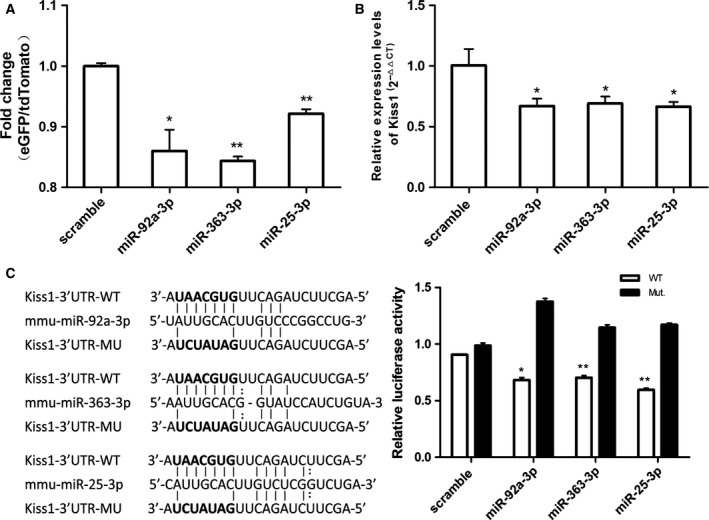
miRNAs affecting the expression of the *Kiss1* gene. (A) miRNA screening by DFRS of *Kiss1* expression (*t* test, every group vs scramble, *P*_miR92a_ = 0.0388, *P*_miR‐363_ = 0.0017, *P*_miR‐25_ = 0.0025, *n* = 3, *P < 0.05, **P < 0.01). (B) qRT‐PCR identified the expression of *Kiss1* in GT1‐7 with miRNA overexpression (*t* test, every group vs scramble, *P*_miR92a_ = 0.1644, *P*_miR‐363_ = 0.1298, *P*_miR‐25_ = 0.1381, *n* = 3). (C) Dual luciferase gene reporter assay for miRNAs targeting the 3′‐UTR of *Kiss1* (*t* test, every group vs scramble, *P*_miR92a_ = 0.0128, *P*_miR‐363_ = 0.0068, *P*_miR‐25_ = 0.019, *n* = 3, *P <* *0.05, **P <* *0.01).

miRNAs are supposed to interfere with protein synthesis by targeting the 3′‐UTR of mRNA 29. Predicting the miRNAs targeting the 3′‐UTR of the *Kiss1* gene using microRNA.org (http://microran.org/microrna/getGeneForm.do), we tested three predicted miRNAs for their inhibitory effects on *Kiss1* expression by the DFRS and their binding efficiency to the 3′‐UTR of *Kiss1* by a dual luciferase gene reporter assay. Significant suppression by miR‐92a‐3p, miR‐363‐3p and miR‐25‐3p of *Kiss1* expression was observed by the reporter system, nearly equivalent to qRT‐PCR and the dual luciferase reporter assay results (Fig. 4).

### Chemical compounds regulating the expression of *Kiss1* gene screened by the DFRS

**Figure 5:**
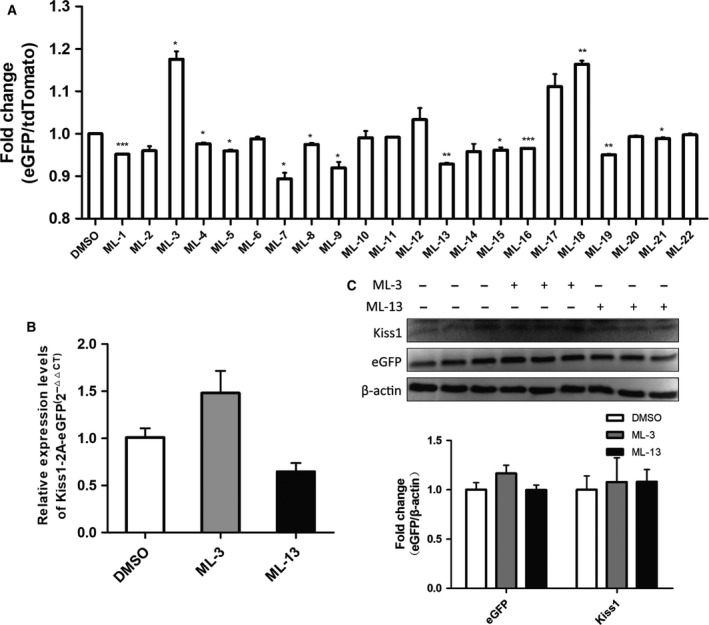
Screening for the *Kiss1* gene expression effectors by DFRS. (A) Chemical compounds screening by DFRS of *Kiss1* expression (*t* test, every group vs DMSO treated group, *P*_ML__‐1_ = 0.0006, *P*_ML__‐2_ = 0.0622, *P*_ML__‐3_ = 0.0114, *P*_ML__‐4_ = 0.0254, *P*_ML__‐5_ = 0.0105, *P*_ML__‐6_ = 0.1992, *P*_ML__‐7_ = 0.0208, *P*_ML__‐8_ = 0.0254, *P*_ML__‐9_ = 0.0272, *P*_ML__‐10_ = 0.5887, *P*_ML__‐11_ = 0.0723, *P*_ML__‐12_ = 0.3355, *P*_ML__‐13_ = 0.0035, *P*_ML__‐14_ = 0.1336, *P*_ML__‐15_ = 0.0133, *P*_ML__‐16_ = 0.0002, *P*_ML__‐17_ = 0.0636, *P*_ML__‐18_ = 0.0023, *P*_ML__‐19_ = 0.0019, *P*_ML__‐20_ = 0.1394, *P*_ML__‐21_ = 0.0152, *P*_ML__‐22_ = 0.6137, *n* = 3, *P <* *0.05, **P <* *0.01, ***P <* *0.001). (B) Relative mRNA expression level of *Kiss1* and eGFP of the reporter treated with ML‐3 and ML‐13 (*t* test, every group vs DMSO treated group, *n *=* *3, *P*_ML__‐1_ = 0.2405, *P*_ML__‐13_ = 0.0788). (C) Relative protein expression level of *Kiss1* and eGFP of the reporter treated with ML‐3 and ML‐13 (*t* test, every group vs DMSO treated group, for eGFP,*P*_ML__‐1_ = 0.3091, *P*_ML__‐13_ = 0.9913; for *Kiss1*,*P*_ML__‐1_ = 0.8568, *P*_ML__‐13_ = 0.7522; *n *= 3). All values shown are means ± SEM.

We used the DFRS to screen chemical compounds affecting *Kiss1* expression. The 22 tested compounds were: ML‐1: sarsasapogenin; ML‐2: senegenin; ML‐3 ~ 15: chalcone derivatives; ML‐16 ~ 18: tanshinone derivatives; ML‐19 ~ 20: isomers of clopidogrel; and ML‐21 ~ 22: tanshinone derivatives. Their molecular masses are in the range 270 ~ 718 Da. Of the 22 compounds, 13 made a significant difference to *Kiss1* expression relative to the control: two of them up‐regulated and 11 down‐regulated *Kiss1* expression (Fig. 5A). We checked the results by qRT‐PCR thereafter, and found only ML‐3 and ML‐13 showed the same pattern as the DFRS consistently (Fig. 5B). Although most of these compounds (ML‐3 ~ 15) share the same carbon skeleton and have similar molecular mass (284.10 ~ 318.07 Da), the small difference caused by the side group of the compounds can be captured by DFRS, but not by qRT‐PCR in the majority of cases. Remarkably, compared with ML‐3, ML‐13 with an additional chlorine atom exerted a contrary effect on *Kiss1* expression. To confirm this difference, we carried out western blot analysis. The results showed the change of eGFP amount remained consistent with that of DFRS detection, while the variation of Kp amount could not be clarified by the western blot due to its lower abundance (Fig. 5C).

## Discussion

We construct a DFRS to monitor the expression of the *Kiss1* gene, with the green fluorescence intensity of eGFP representing the activity of transcription and translation of *Kiss1*, and the red fluorescence intensity of tdTomoto normalizing the non‐specific factors affecting the protein synthesis in the cell. Through homology directed repair‐mediated targeting with the help of CRISPR/Cas9 technology, an eGFP gene was inserted into the open reading frame of the *Kiss1* gene, and then transcribed and translated together with the *Kiss1* gene, regulated by the full set of regulatory elements of the *Kiss1* gene. Therefore, any fluctuation of *Kiss1* expression can be visualized with a fluorescence detector, which made possible rapid and high throughput screening for effectors affecting *Kiss1* expression. Moreover, the tdTomoto gene, used as an internal control, was added to the system by the neo‐diphtheria toxin A, positive–negative selection targeting method. The internal control is indispensable for relative quantitative detection. In quantitative real time PCR detection, for example, housekeeping genes are usually used as internal controls because their share of total RNA remains constant in different physiological states, in both slowly and actively proliferating cells, and in apoptotic cells. Having been normalized by internal control(s), the product amount of a specific gene, which is differentially regulated in response to divergent external stimuli, can be compared among samples treated differently 30. In our DFRS, tdTomoto was used as a visible ‘housekeeping gene’, as it maintained stable expression when external stimuli, such as E_2_, rapamycin and miR‐363‐3p, specifically influenced the expression of *Kiss1*. However, when certain external factors, such as some chemical compounds in our test, were cellularly toxic or affected cell proliferation and viability, both eGFP and tdTomoto could suffer. With the internal control, non‐specific influences on *Kiss1* expression can be discriminated from specific ones. Having been normalized against the internal control, the specificity and the accuracy of the comparative results can be guaranteed.

Several laboratory instruments can be applied to detect the fluorescence intensity quantitatively, including a fluorospectrophotometer, microplate reader and flow cytometer. We tried to collect the fluorescence intensity data on a fluorescence microplate reader at first, which was capable of obtaining 96 items of data at one time in 1 min. Unfortunately, the modest differences between samples were hardly able to be evaluated statistically with the limited number of duplicates. The benefit of flow cytometry is that it analyses the fluorescence intensity of cells one by one, that is it collected 10^4^ items of data for one sample with 10^4^ cells. Given that the fluorescence intensity value of one sample is the mean of 10^4^ items of data, the mild but significant difference between samples can be readily confirmed.

We applied the DFRS with flow cytometry to validate the effect of some canonical regulators of the *Kiss1* gene at first, and their respective stimulation or repression characteristics were in line with expectation, except for rapamycin. Rapamycin was reported to lower the mRNA level of *Kiss1* in rat hypothalamus 21, but we found the mRNA amount of *Kiss1* and eGFP increased in rapamycin‐treated GT1‐7 cells by qRT‐PCR, while the Kp protein decreased in the same cell detected by both western blot and flow cytometry, which was consistent with the results that the increase of Kp protein could be induced by activated mTOR signaling in rat preoptic area/AVPV tissue 31. Rapamycin is a specific inhibitor of mTOR signaling that participates in the regulation of protein synthesis by phosphorylating its downstream targets, S6K1 and 4EBP1 32. Over past years, cumulative evidence has demonstrated that mTORC1 (the direct target of rapamycin) inhibition, in addition to reducing protein synthesis, deeply affects gene transcription by influencing the activity of some transcription factors 32, 33. In some lung cancer cell lines, rapamycin was proved to result in increased *Pdcd4* mRNA level through *cis*‐acting element(s) located in the 5′‐flanking region of this gene 34. In the work of Roa *et al*. 21, central rapamycin administration caused *Kiss1* mRNA to decrease in whole hypothalamic preparations from ovariectomy + E_2_ rats, yet the magnitude of inhibition differed between ARC and AVPV by *in situ* hybridization, which implied distinct mechanisms (direct vs indirect) of *Kiss1* mRNA decrease in the two regions. The contradictory role of rapamycin on the transcriptional and translational level of regulation for the *Kiss1* gene obtained in GT1‐7 cells has expanded our knowledge of the complications of mTOR signaling.

The DFRS provides a scheme to detect a protein of interest quantitatively. Compared with the two most popular quantitative protein methods in the laboratory, western blot and ELISA, which both require procedures of sample preparation, primary and secondary antibody incubation, and color development with a chromogenic reagent, the DFRS is time and cost effective, and is able to do multiple screenings. By flow cytometry detection, the DFRS is able to provide a sensitive and accurate comparison between multiple factors affecting *Kiss1* expression at one time.

We investigated the capacity of DFRS for multiplexing with 22 chemical compounds, most of them natural products. Thirteen of them showed a significant difference from the control in regulating *Kiss1* gene expression. The smallest variance with statistical significance between samples was as low as 1.2% (ML‐21 vs control) by DFRS detection, which was nearly impossible for qRT‐PCR or western blot to accomplish. When we tried to validate the results by the real time qRT‐PCR assay, we found some of the RNA samples extracted from the chemically treated cells were not able to give stable results, probably because of the toxicity of the compounds, inducing RNA degradation before cell lysis. Moreover, variation in the mRNA amount of a gene did not parallel the change of protein amount under some circumstances, for instance RNA interference or miRNA targeting. Therefore, the protein‐based DFRS may be a better choice to evaluate the fluctuation in expression of a protein‐coding gene.

Although the system has many advantages in effector screening, some issues should be considered when the screening results need to be evaluated carefully. For instance, the immortalized GnRH neuron cell line GT1‐7 employed in this study cannot duplicate the GnRH neurons completely, as *in vivo* the majority of GnRH neurons do not express the *Kiss1* gene, except for some fetal GnRH neurons 35. Therefore, the responses in the GT1‐7 cells to environmental factors might not parallel those under normal physiological situations entirely. In addition, given that the half‐life of eGFP could be different from that of Kp, the fluorescence intensity of eGFP is more appropriate to represent the activity of transcription and translation of *Kiss1* rather than the dynamic state of the Kp protein.

In conclusion, the advantages of DFRS detection include the following: it is a protein‐based method, avoiding some defects of RNA‐based technologies; it does not depend on a specific antibody, with better sensitivity and accuracy; and it has high multiplexing capability. Its potential application in gene functional analysis, drug screening and pharmaceutical study can be expected in the future.

## Author contributions

XL performed experiments, analyzed data and wrote the manuscript. YZ designed experiments and wrote the manuscript. SW performed experiments. YL, JX and KL designed experiments. HY and LM designed and synthesized the chemical compounds. All authors approved the final manuscript.
